# A Heartbeat Classifier for Continuous Prediction Using a Wearable Device

**DOI:** 10.3390/s22145080

**Published:** 2022-07-06

**Authors:** Eko Sakti Pramukantoro, Akio Gofuku

**Affiliations:** 1 Graduate School of Interdisciplinary Science and Engineering in Health Systems, Okayama University, 3-1-1 Tsushimanaka, Kita-Ku, Okayama 700-8530, Japan; 2 Faculty of Computer Science, Brawijaya University, Malang 65145, Indonesia

**Keywords:** heartbeats, machine learning, deep learning, wearable sensor

## Abstract

Heartbeat monitoring may play an essential role in the early detection of cardiovascular disease. When using a traditional monitoring system, an abnormal heartbeat may not appear during a recording in a healthcare facility due to the limited time. Thus, continuous and long-term monitoring is needed. Moreover, the conventional equipment may not be portable and cannot be used at arbitrary times and locations. A wearable sensor device such as Polar H10 offers the same capability as an alternative. It has gold-standard heartbeat recording and communication ability but still lacks analytical processing of the recorded data. An automatic heartbeat classification system can play as an analyzer and is still an open problem in the development stage. This paper proposes a heartbeat classifier based on RR interval data for real-time and continuous heartbeat monitoring using the Polar H10 wearable device. Several machine learning and deep learning methods were used to train the classifier. In the training process, we also compare intra-patient and inter-patient paradigms on the original and oversampling datasets to achieve higher classification accuracy and the fastest computation speed. As a result, with a constrain in RR interval data as the feature, the random forest-based classifier implemented in the system achieved up to 99.67% for accuracy, precision, recall, and F1-score. We are also conducting experiments involving healthy people to evaluate the classifier in a real-time monitoring system.

## 1. Introduction

A heart disease that leads to life-threatening situations can be prevented by conducting regular heartbeat condition monitoring [1]. For early detection of heart disease, the common procedure is to conduct a heartbeat measurement using an electrocardiogram (ECG). Equipment such as a Holter monitor is utilized to obtain ECG data. Next, the physician will analyze the recording to seek the pattern regarding abnormality patterns. Conducting regular checkups can be challenging due to non-technical and technical aspects. An example of the non-technical aspect is a pandemic situation that leads to difficulties in making an appointment with a physician or other things such as busyness. The technical aspect is related to the technology for conducting a regular checkup. Recording a cardiac activity using a Holter monitor has a drawback that limits the patient’s activity, especially for long-term recording. In some cases, it is necessary to conduct a long-term recording of ECG because the irregular heartbeat may not appear during short examinations in health care facilities. For this case, flexible ECG equipment is preferred. Moreover, interpreting a long electrocardiogram recording will burden medical staff. Thus, an automated ECG analysis is needed.

Currently, flexible ECG equipment is available as a wearable devices such as chest traps, fitness devices, smartwatches, or armbands. Initially, those devices are intended for fitness equipment. Nonetheless, a chest strap such as Polar H10 can replace a Holter monitor to record cardiac activity [2]. This device is better than a Holter monitor for measuring the RR interval of a person’s heart rate and RR interval while they are moving, running, cycling, swimming, and other activity at the gym [3]. A coin battery powers it for up to 30 h of active usage. While being used on one’s chest, it does not affect one’s movement. Polar H10 is also equipped with the Bluetooth Low Energy (BLE) to interact with other equipment [4]. It produces several formats of cardiac parameters such as heart rate (HR), RR interval (RRi), and electrocardiography (ECG) [5]. Among those parameters, only the RRi is suitable for real-time and continuous detection of a heartbeat using Polar H10. Polar H10 sends RRI data every second in a fixed amount, while the value of ECG data fluctuates. Thus, ECG data cannot be used in real-time prediction because such data should be recorded in batches before processing.

Previously, we have investigated that HR and RRi data from Polar H10 can be sent every second through Bluetooth Low Energy [6]. Using RRi produced by Polar H10 as a feature for heartbeat classification opens opportunities to develop real-time and continuous heartbeat monitoring. Besides, related studies have proposed heartbeat classifiers by combining several features, namely RRi, wavelet, ECG morphology, and heart rate variability (HRV) with machine learning algorithms to achieve higher classification accuracy [7]. RRi data can be extended into HRV features and RRi series such as local RRi and normalized RRi. HRV can be used as a feature for automated heartbeat classification; however, it will lead to binary classification, such as normal and abnormal decisions. As shown in [8,9], they used HRV as a classification feature to distinguish between a normal and an anomaly event. Using the RRi series as a feature for classification provides more detail to classify the types of heartbeats instead of normal and abnormal events. Additionally, there are still limited developments in real-time predicting a heartbeat sequence using commercial wearable devices.

In this study, we developed a real-time and continuous heartbeat monitoring system using a commercial wearable device. Polar H10 is employed to produce RR interval continuously. We chose Polar H10 because it can produce a gold standard cardiac sign [2]. Previous studies that used polar H10 were focused on the heart rate variability (HRV) measurement [10,11]. HRV can be used as a feature for classification, but it is limited to the normal and abnormal conditions [8]. Compared to the previous study, we presented a system using this device to provide a more detailed heartbeat prediction, namely, normal beat (N), supraventricular ectopic beat (SVEB), ventricular ectopic beat (VEB), fusion beat (F), and unknown beat (Q), following the described classes by the Association for the Advancement of Medical Instrumentation® (AAMI). While other studies combine several features to achieve higher accuracy [7], our study presents all possibilities in training a classifier to achieve higher accuracy using only the RRi features. Moreover, the classifier should give a prediction result in less than one second following the received data from Polar H10 that are sent every second. Furthermore, we train our classifier using machine learning and deep learning methods on inter-patient and intra-patient schemes of the MIT-BIH arrhythmia database [12]. The MIT-BIH arrhythmia database is a well-known database. However, the classes in this database are imbalanced. To overcome this issue, we applied oversampling methods [13] to achieve higher classifier accuracy. The experiment shows that it increased the accuracy up to 99.67%. We implemented the classifiers in our framework to evaluate their performance in providing real-time prediction of a healthy person every second. As a result, all classifiers can perform in less than one second. We also demonstrate our study with several participants. The contribution of this study is the proposal of a continuous heartbeat monitoring system using Polar H10 as a cardiac sensor and shows all possibilities of creating a heartbeat classifier based on RRi as the only classification feature. Thus, our study offers advanced experiments on heartbeat classification compared to other studies.

## 2. Automated Heartbeats Classification

Heart disease can be recognized according to the heartbeat characteristics on an ECG recording where the pattern correlates with the heart condition’s state. Usually, medical experts will determine the state of a patient’s heart condition by the shape or morphology of the ECG waves. However, manually determining the pattern is challenging and laborious for professionals, especially for long ECG recordings. Moreover, the human eye can be inappropriate for detecting the morphological variation of the ECG waves. Thus, the use of computational techniques for automatic classification is needed.

The benefit of an automated heartbeat classifier combined with a wearable heart sensor device enables the real-time detection of abnormalities in our heartbeats. The Association for the Advancement of Medical Instrumentation ${}^{®}$ (AAMI) defines heartbeats into five classes [14]. As shown in Table 1, those beats are categorized as normal (N), supraventricular ectopic beat (SVEB), ventricular ectopic beat (VEB), fusion beat (F), and unknown beat (Q). Among them, SVEB and VEB are categorized as problems in our heart condition, where VEB is related to heart failure [15] and SVEB is related to atrial fibrillation [16].

A comprehensive survey on heartbeat classification using machine learning was presented by Luz [7] while another study using deep learning was presented by Ebrahimi [17]. One of the differences between classification using machine learning and deep learning methods is the feature that is extracted. Deep learning offers automatic feature extraction, while machine learning mainly uses the handcrafted feature. The reports of automatic heartbeat classification are varied. Some use different classes and databases, thus leading to unfair comparison—unfortunately, only a few follow AAMI recommendation [18]. The Automated heartbeat classification requires several features to distinguish between normal and abnormal beats. Those features are extracted from electrocardiography recordings, such as the RR interval series, the morphology of ECG waves, and wavelets. After that, a machine learning or deep learning method was used as a classifier.

Lin [19] explored the combination of a normalized RR interval and morphological ECG waves as features. They used the linear discriminant to classify normal, supraventricular, and ventricular beats. As a result, normalized RR intervals increase the classifier’s performance. Tsipouras uses three RRi features (R1, R2, and R3); thus, the rule-based and deterministic automation is used to classify normal, premature ventricular contraction, ventricular flutter/fibrillation, and two heart blocks [20]. Lian uses a method to map RR intervals to detect atrial fibrillation [21]. Xiang uses CNN as feature extraction to obtain time intervals between two RR intervals and morphological features as one-dimensional data, thus using a multi-layer perceptron (MLP) to classify VEB and SVEB [22]. Sannino uses RR interval features consisting of previous RR, post RR, local average within 10 s slidings from the previous window, and average 10 RR interval window within 5 min. They use ANN as a binary classifier to predict normal and abnormal beats [23]. Ankita uses R-peak and RR interval as a feature and uses hybrid CNN to classify 16 classes of heartbeat [24]. Jose did an investigation of feature selection for heartbeat classification. He suggests that using normalized RR intervals could increase the classifier’s performance [25]. Mondejar demonstrates using several features such as RR interval, normalized RR interval, high order statistic, HBF coefficients, and wavelet transform, thus using a support vector machine (SVM) to classify each feature [26]. Developing automatic heartbeat classification systems on resource-constrained devices is challenging, e.g., discovering an optimal mixture of features and classifiers [25].

## 3. Materials and Methods

### 3.1. Dataset and Features

This study uses a dataset from the MIT-BIH arrhythmia database [12]. Even though this dataset is imbalanced (imbalanced data would impact classification accuracy), these data have already been labeled, annotated, and are publicly available. The dataset consists of 48 recordings of patient’s data. Each datum has a 30-min ECG recording. Among 48 recording numbers, 102, 104, 107, and 217 are omitted for training data because they consist of paced rhythm. Furthermore, we extract features for classification using this database. In this study, the feature used for training the classifier is adapted to the sensor output data types: RR interval and ECG data. RR interval data are measured from the distance of the two R peak in each ECG wave (PQRS). This variable can reflect the physical condition [11]. Detecting the R wave in the ECG recording is needed to calculate the RR interval. In this case, we used Pan-Tompkins Algorithm [27] to calculate the distance from one R wave to the next detected R wave. After the RR interval’s value is known, we calculate the RR interval series as one feature within 42 windows of RR interval data. There are several types of RR interval series, as shown in Table 2. We extract the RR interval series as a feature from the training and testing data. The RR interval series has the following characteristics: RR0, RR-1, RR+1, RR0/avgRR, RR-1/avgRR, RR-1/RR0, RR-1/RR0, RR+1/avgRR, RR+1/RR0. An average RR interval in the period window is required to calculate a normalized RR interval. Usually, the average RR interval is calculated in a patient-wise way. Patient-wise means calculating the average RR of all recorded data. In a real-time scenario, especially in stream processing, the calculation of entire recorded data is impossible because data keep growing. Thus it is suggested to compute previously known data. In this study, feature extraction uses 42 previous RR intervals to minimize computational time and to speed up the classification process. For this reason, for feature extraction for training the classifier from the MIT-BIH arrhythmia database, the average RR interval is calculated from 42 windows of the previous RR interval. The RR interval can be computed into nine features; thus, it does not need feature selection due to its low complexity.

### 3.2. Oversampling

**Figure 1:**
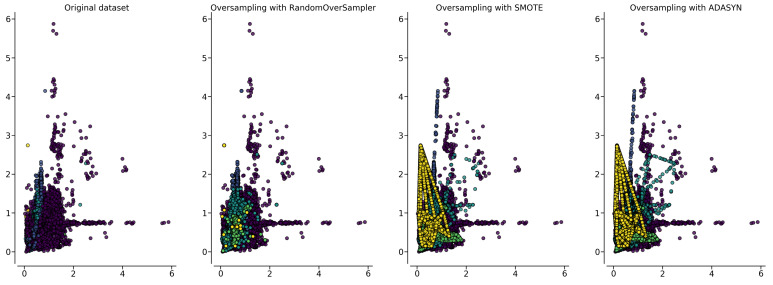
Plotting data of RR interval feature.

As shown in Table 3, the original data of the MIT-BIH arrhythmia database are dominated by the N class, while the number of class Q instances is only 15. The imbalanced dataset will affect the performance of a classifier. Thus, we applied oversampling methods such as Random Oversampling (ROS), the Synthetic Minority Oversampling Technique (SMOTE), and Adaptive Synthetic Sampling (ADASYN). A Python library was used to balance the dataset using RandomOverSampler, SMOTE, and ADASYN [13]. Random Oversampling works by duplicating the minority class until sample data become equal to those of the majority class. SMOTE and ADASYN work by generating new values by using the rule given by Equation (1), where $x_{new}$ is a newly generated value between k nearest-neighbors of $x_{i}$ and $x_{zi}$. In contrast, λ is a random number between zero and one. ADASYN generates data proportionally regarding the number of the majority class in $x_{i}$ period. Figure 1 shows a visual representation of the first two features of nine RR interval features on a different class. There are five colors representing each class. In the first plot, a yellow dot represents the minority class. In the next plot, yellow dots are multiplied using the oversampling method. We can see the behavior of oversampling method to balance the dataset. The balanced dataset is shown in Table 3. Using the oversampling method, the the number of data on the minority classes are nearly equal to those of the majority class. The number of data which belongs to the majority class is 90,125. Using random oversampling and SMOTE, the numbers of data in all classes are equaled, while using ADASYN, several minority classes exceeded the amount of N classes.

(1) $$x_{new}=x_{i}+\lambda \times \left(x_{zi}-x_{i}\right)$$

### 3.3. Train the Classifiers

We train the classifier using inter-patient and intra-patient paradigms with the MIT-BIH arrhythmia dataset to create the best classifier based on those features. The inter-patient paradigm means that the training and testing data come from different patient recordings. Later, it is called protocol splitting because many previous studies used this method to split the training and testing data [28]. At the same time, in the intra-patient paradigm, the data for training and testing may come from the same patient recording, which later is called random splitting. The protocol splitting will make the classifier work harder because the model will classify new data [7]. The splitting data based on inter-patient data are defined as follows, training dataset using record number: 101, 106, 108, 109, 112, 114, 115, 116, 118, 119, 122, 124, 201, 203, 205, 207, 208, 209, 215, 220, 223, 230 and testing dataset using record number: 100, 103, 105, 111, 113, 117, 121, 123, 200, 202, 210,212, 213, 214, 219, 221, 222, 228, 231, 232, 233, 234. While in intra-patient, the scheme of splitting data is undertaken randomly, selecting 70% from available data as training data and the remaining as testing data.

Several machine learning and deep learning methods are used to classify five classes of heartbeats for classification methods. We use Scikit learn library in Python to train the model using Decision Tree (DT), Gradient Boosting (GB), k-Nearest Neighbors (KNN), Multi-layer Perceptron (MLP), Random Forest (RF), and Support Vector Machine (SVM). The training parameter are shown in Table 4.

For deep learning, we use tensor flow to train the model using sequential with artificial neural networks (ANN) [29]. A summary of the model is shown in Table 5. There are seven layers with nine nodes at the input layer, five nodes at the output layer, and five hidden layers. The activation function is relu and softmax, kernel regularizer (l2) is 0.0001, the optimizer is adam, and the loss function is sparse categorical cross-entropy. Four evaluation metrics such as accuracy, precision, recall, and F1-score is used to evaluate the classifiers.

(2) $$Accuracy=\frac{TP+TN}{TP+TN+FP+FN}$$

(3) $$Precision=\frac{TP}{TP+FP}$$

(4) $$Recall=\frac{TP}{TP+FN}$$

(5) $$F1\text{-}score=\frac{2\times Precision\times Recall}{Precision+Recall}=\frac{2\times TP}{2\times TP+FP+FN}$$

Evaluation is performed by validating the model with data testing. The accuracy is a metric to measure the correctness of the predicted class with the true class in the dataset. The precision parameter defines a correct prediction class divided by all numbers resulting from prediction or known as the positive predicted value. At the same time, recall is used to measure the actual value of the predicted class that is identified correctly or known as sensitivity. The F1-score measures the balance between precision and recall, especially in the imbalance dataset. For the first model, we use several machine learning techniques to train a classifier by splitting the data using a protocol from [28] and a random split as an intra-patient paradigm. For the intra-patient training and testing data, we split randomly from the whole recording by 70% for training and 30% for testing. As shown in Table 6, we have three kinds of data splitting mechanisms. The first one is protocol split, the second is random split, and the third is random split of over-sampled data. Thus, we are conducting the training for those splitting for each classification method. We performed training five times to validate the result for random splitting.

## 4. Results

**Figure 2:**
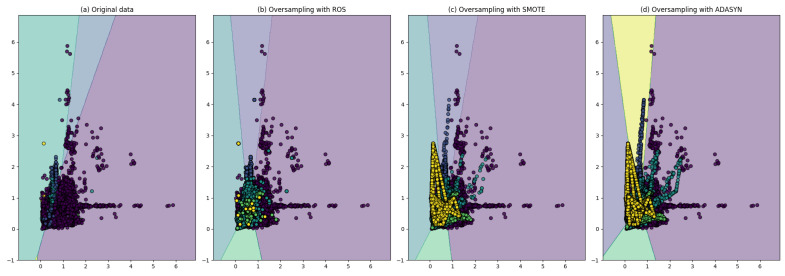
Decision boundary using logistic regression.

We conducted three schemes for training the classifier based on the dataset splitting scheme. The first scheme uses the inter-patient splitting, and the second scheme uses the intra-patient with a random split on the original dataset. The third scheme is intra-patient with a random split on the over-sampled dataset. The result of the first training is shown in Table 7. The highest accuracy was achieved by an SVM-based classifier with 92.57% and 90.23%, 92.57%, 90.81% for precision, recall, and F1-score, consecutively. While Neural Network-based classifier achieved the accuracy of 92.50% and 91.36%, 92.50%, 91.46% for precision, recall, and F1-score, consecutively. As a supplement for those results, we present the confusion matrix at Table 8 and Table 9, where the horizontal value is the result of prediction by the classifier and the vertical is an actual label. As we can see in the confusion matrix, the result is not so good, several values are predicted in the wrong class, and both the classifiers cannot predict the Q class (the Q class is predicted as the N class). This result is caused by many overlapping data features with other classes, as we can see in Figure 2 with original data, i.e., minority class (with the yellow dot appears inside another class). As stated by [28] the way of data splitting will burden the classifier, especially with imbalanced data.

The second training was conducted using a random dataset split with 70% for training and 30% for testing. The training and testing were performed in five times repetition. The ANN-based classifier achieved the highest accuracy with 96.25% and 96.07%, 96.35%, 96.09% for precision, recall, and F1-score. As shown in Table 10, Random Forest-based classifier yields 96.22% accuracy with 95.94%, 96.21%, 95.89% for precision, recall, and F1-score, respectively. Based on the confusion matrix shown in Table 11 and Table 12.

There is still a miss-match by the classifier to predict actual label. Overall the result of the accuracy of each classifier is better than the protocol split. The minority class (Q) by the classifiers based on inter-patient and intra-patient are classified as a normal class, and several works reported skipping the minority class and focusing on classifying the N, S, and V class [7].

The third training was conducted by an intra-patient scheme using over-sampled data by Random Oversampling, SMOTE, and ADASYN. The number of data for training is 315,437 and 135,188 for testing data. In this configuration, the amount of data for each class is nearly equal. As a result, the maximum accuracy achieved is 99.67% by the Random Forest-based classifier. The precision, recall, and F1-score are 99.67%, 99.67%, and 99.67%. The second highest accuracy is the Decision Tree classifier with 99.31%, 99.32%, 99.31%, 99.31% for accuracy, precision, recall, and F1-score, respectively. Table 13 shows the result of all classifiers using a third training scheme. Based on the oversampling method, Random oversampling is dominant compared to other oversampling methods in terms of classifier accuracy. The way the ROS works by duplicating the minority class may lead to this dominance. However, the classifier trained using SMOTE also gives good results that achieves 98.15% accuracy by the random forest classifier. As shown in confusion matrix Table 14, Table 15 and Table 16, the overlap causing miss-prediction by the classifier is fewer compared to the confusion matrix based on training classifiers using scheme one and two. These classifiers can recognize the F and Q classes, while the classifier based on training one and two schemes failed to predict the F and Q classes.

Table 17 shows the comparison of our classifier with previously proposed classifiers. The trained classifier in this study has competitive performance among previously reported classifiers. Moreover, our classifier only uses a simple feature from the RR interval series. Some classifiers can achieve higher accuracy compared to those previously reported.

## 5. Real-Case Experiment

**Figure 3:**
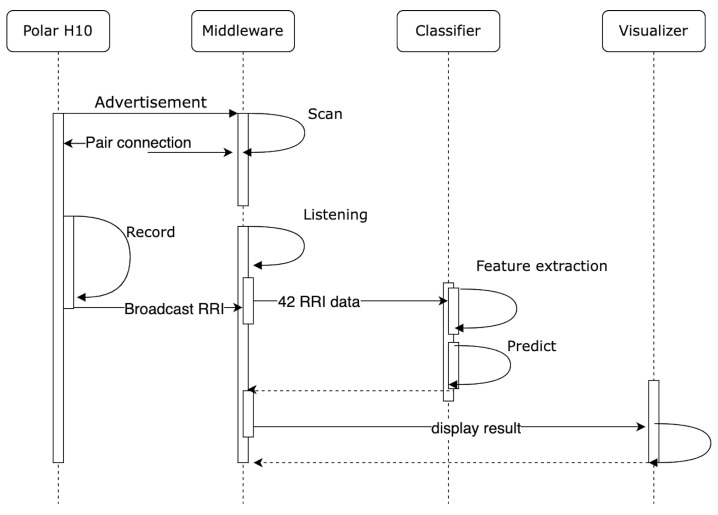
Class diagram of real-time monitoring system.

**Figure 4:**
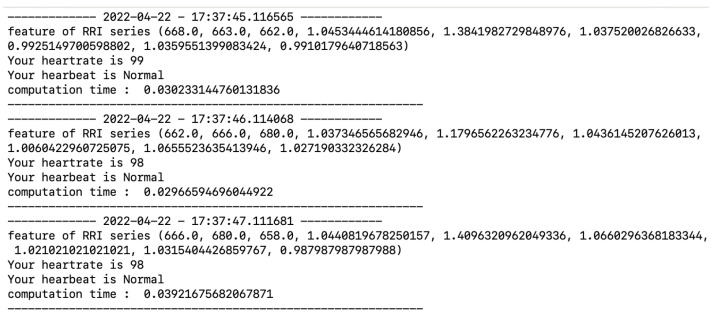
An output of real-time prediction of heartbeat.

In this section, we provide an experiment using our classifier and our developed system to continue the monitoring of heartbeat in real-case scenarios. This experiment was conducted by involving a healthy person to measure the capabilities of a classifier to predict data continuously and as a preliminary experiment to validate our developed system. We choose the classifier with accuracy above 96% for each method among all the classifiers. The experiment runs for 20 min for each classifier. As shown in Figure 3, our experiment uses Polar H10 as a sensor, middleware, classifier, and visualizer. The middleware, classifier, and visualizer are run on a personal computer. The application works as follows: (1) The middleware initiate communication through BLE with Polar H10. In this study, we use BLEAK as the BLE framework. Our previous study concluded that Polar H10 and middleware could maintain good communication by receiving signal strength (RSSI) above −80 dBm until 50 m at no obstacle environment and 16 m at obstacle environment [4]. (2) After communication has been made, middleware requests heart rate measurement. (3) Polar H10 will send data by broadcast, consisting of RR interval and heart rate. (4) The middleware will listen until it receives 42 RR interval data. The classification process will start if 42 RR interval data are collected. (5) The classification process is started with feature extraction to form nine kinds of RR interval series. (6) The classifier predicts RR interval series to determine the class. (7) The prediction result is visualized in the command line interface (CLI), as an example can be seen in Figure 4. Figure 4 consists of information regarding the time of recording, extracted feature, heart rate, prediction result, and computation time after the run-time of the application reached 20 min, then middleware closing connection with Polar H10.

The performance of the classifier is presented in Table 18. Classifiers based on Random Forest have the longest average processing time with 0.108851 s. The classifier with the fastest processing time is the Decision Tree with 0.00035943 s. During 20 min, the number of beats varies, and most of the prediction results are normal beats. The average inference time of the classifiers is less than 1 s, and they can give prediction results within one second. Thus, the classifier is suitable for the continuous and real-time prediction of a heartbeat. We also provide information regarding RSSI during the experiment. As shown in Table 18, the average RSSI is above −80 dBm, which indicates that the transmission data between sensor and middleware are in good condition while the participant moves around the middleware.

We also conducted an experiment involving six healthy people to evaluate our developed system; four participants are male, and two are female. Their ages also varied. We used a random forest classifier trained with random oversampling in this experiment. The experiment runs for 30 min for each participant. We also measured the received signal strength indicator (RSSI) for the quality of received data from Polar H10. As we can see in Table 19, the number of beats in 30 min from each participant is varied. All of the received beats are predicted as normal. According to the RSSI, we can conclude the transmission data are in a good state, which is above −80 dBm. The value of RSSI also indicates the distance between the participant with the middleware device. The more excellent value of RSSI means the participant is closed to middleware.

## 6. Conclusions

This study presents a heartbeat classifier based on RR interval as a real-time and continuous heartbeat monitoring feature. Several machine learning algorithms were explored to classify the well-known MIT-BIH arrhythmia database. The imbalance classes problem of the dataset is addressed by implementing oversampling methods. As a result, a random forest-based classifier on the over-sampled data performed best by 99.67% for accuracy, precision, recall, and F1-score. Furthermore, we evaluate the classifier on our framework. The first evaluation continuously predicts the heartbeat of a healthy person to measure prediction time in a real-time scenario. As a result, all the classifiers can predict the data in under 1 s. Thus, it can be concluded that the classifiers are suitable to predict Polar H10 data output in a continuous and real-time manner. In the second evaluation, we increase the number of participants to four males and two females. Their age is varied and in the healthy condition. The result is our system predicts their heartbeat as normal, and transmission data between Polar H10 and middleware is in a good state, indicated by RSSI above −80 dBm.

In the future, we would like to extend the implementation for real experimental studies in corporation with a medical professional to identify the type of heart disease and other real-case scenarios where users perform more vigorous activities, such as sports.

## Figures and Tables

**Table 1:** AAMI recommendation of heartbeats.

Normal (N)	Supraventricular Ectopic Beat (SVEB)	Ventricular Ectopic Beat (VEB)	Fusion Beat (F)	Unknown Beat (Q)
Normal beat (N)	Atrial premature beat (A)	Premature ventricular contraction (V)	Fusion of ventricular and normal beat (F)	Paced beat (/)
Left bundle branch block (L)	Aberrated atrial premature beat (a)	Ventricular escape beat (E)		Fusion of paced and normal beat (f)
Right bundle branch block (R)	Nodal (junctional) premature beat (J)			Unclassified beat (Q)
Atrial escape beat (e)	Supraventricular premature beat (S)			
Nodal junctional escape beat (j)				

**Table 2:** RR interval feature series.

Features Series	Descriptions
RR0	Current RRi value
RR-1	Previous RRi value
RR+1	Next RRi value
RR0/avgRR	Current RRi/average of RRi within 42 s
tRR0	(CurrentRR-averageRR)/stddevRR
RR-1/avgRR	Previous RRi/average of RRi
RR-1/RR0	Previous RRi/ current RRi within 42 s
RR+1/avgRR	Next RRi, average of RRi within 42 s
RR+1/RR0	Next RRi, current RRi

**Table 3:** Distribution of heartbeats class in MIT-BIH data.

	Original	ROS	SMOTE	ADASYN
number of N	90,125	90,125	90,125	90,125
number of S	2781	90,125	90,125	90,332
number of V	7009	90,125	90,125	89,215
number of F	803	90,125	90,125	90,293
number of Q	15	90,125	90,125	90,120

**Table 4:** Model parameters.

Model	Parameter
DT	default
GB	estimator = 100, learning rate = 0.1, max. depth = 3, random state = 0.
kNN	k = 3.
MLP	network solver = adam, alpha=1e-5, hidden layer = 128, input layer = 9 output layer = 5, max iteration = 600, random state = 42.
RF	tree = 30, random state = 42.
SVM	kernel = RBF, gamma = 0.8, C = 1.

**Table 5:** Deep learning model summary.

Layer (Type)	Output Shape	Param
dense (Dense)	314,857, 9	80
dense_1 (Dense)	314,857, 64	576
dense_2 (Dense)	314,857, 128	8320
dense_3 (Dense)	314,857, 512	66,048
dense_4 (Dense)	314,857, 128	65,664
dense_5 (Dense)	314,857, 64	8256
dense_6 (Dense)	314857, 5	325

**Table 6:** Splitting the data.

	Protocol Split		Random Split		Oversampling	
	Train	Test	Train	Test	Train	Test
number of N	45,866	44,259	63,150	26,975	63,050	27,075
number of S	944	1837	1973	808	63,225	26900
number of V	3788	3221	4845	2164	63042	27,083
number of F	415	388	536	267	63,076	27049
number of Q	8	7	9	6	63,044	27,081
Total	51,021	49,712	70,513	30,220	315,437	135,188

**Table 7:** Result of machine learning using protocol split.

Method	Accuracy (%)	Precision (%)	Recall (%)	F1-Score (%)
DT	89.15	88.30	89.15	88.64
GB	89.08	89.10	89.08	88.44
KNN	90.76	88.42	90.76	89.42
NN	92.50	91.36	92.50	91.46
RF	91.81	89.24	91.81	90.29
SVM	92.57	90.23	92.57	90.81
ANN	91.44	88.59	91.04	89.72

**Table 8:** Confusion matrix SVM.

Classifier						
Reference		**n**	**s**	**v**	**f**	**q**
	N	43,588	49	622	0	0
	S	1159	79	599	0	0
	V	808	64	2349	0	0
	F	385	0	3	0	0
	Q	6	1	0	0	0

**Table 9:** Confusion matrix NN.

Classifier						
Reference		**n**	**s**	**v**	**f**	**q**
	N	43,170	199	872	18	0
	S	789	279	768	1	0
	V	619	55	2535	12	0
	F	382	0	5	1	0
	Q	6	0	1	0	0

**Table 10:** Result of machine learning using random split dataset.

Method	Accuracy (%)	Precision (%)	Recall (%)	F1-Score (%)
DT	94.08	94.12	94.08	94.10
GB	95.57	95.29	95.57	95.21
KNN	95.08	94.50	95.08	94.53
NN	95.82	95.53	95.82	95.46
RF	96.22	95.94	96.21	95.89
SVM	95.05	93.97	95.05	94.35
ANN	96.35	96.07	96.35	96.09

**Table 11:** Confusion matrix RF.

Classifier						
Reference		**n**	**s**	**v**	**f**	**q**
	N	26,734	20	206	15	0
	S	129	594	85	0	0
	V	420	43	1698	3	0
	F	211	0	6	50	0
	Q	6	0	0	0	0

**Table 12:** Confusion matrix ANN.

Classifier						
Reference		**n**	**s**	**v**	**f**	**q**
	N	26,776.2	28	214.6	15.2	0
	S	112.8	632.6	88.6	0	0
	V	392.6	44.6	1663	10.8	0
	F	178	0	9.4	46.6	0
	Q	5.75	0.75	0.5	0	0

**Table 13:** Result of training using over-sampled dataset.

	Accuracy (%)			Precision (%)			Recall (%)			F1-Score (%)		
	R	S	A	R	S	A	R	S	A	R	S	A
DT	**99.31**	96.50	96.08	99.32	96.49	96.07	99.31	96.50	96.08	99.31	96.49	96.06
GB	89.57	86.73	78.26	89.62	86.70	77.94	89.57	86.73	78.26	89.55	86.67	78.01
KNN	**98.93**	97.55	97.49	98.99	97.71	97.56	98.97	97.68	97.49	98.96	97.64	97.44
NN	89.88	90.06	84.48	90.17	90.19	84.54	89.88	90.06	84.48	89.81	89.96	84.23
RF	**99.67**	98.15	98.08	99.67	98.15	98.09	99.67	98.15	98.08	99.67	98.14	98.07
SVM	87.87	87.43	79.83	87.93	87.46	79.59	87.87	87.43	79.83	87.78	87.32	79.39
ANN	**97.51**	96.20	95.85	97.54	96.22	95.81	97.51	96.20	95.85	97.49	96.18	95.83

**Table 14:** Confusion matrix RF-ROS.

Classifier						
Reference		**n**	**s**	**v**	**f**	**q**
	N	26,626	32	370	44	2
	S	0	26,900	0	0	0
	V	0	0	27,083	0	0
	F	0	0	0	27,049	0
	Q	0	0	0	0	27,081

**Table 15:** Confusion matrix DT-ROS.

Classifier						
Reference		**n**	**s**	**v**	**f**	**q**
	N	26,148	141	576	205	5
	S	0	26,900	0	0	0
	V	0	0	27,083	0	0
	F	0	0	0	27,049	0
	Q	0	0	0	0	27,081

**Table 16:** Confusion matrix ANN-ROS.

Classifier						
Reference		**n**	**s**	**v**	**f**	**q**
	N	24,773.4	255.8	943.4	1073.6	27.8
	S	84.2	26,670.75	146.5	2.4	0
	V	334.4	123.8	26,277.75	310.4	0.4
	F	50	0	13.6	26,992	0
	Q	0	0	0	0	27,082

**Table 17:** Works comparison following AAMI recommendation.

Classifier	Feature	No. of Features	Data Scheme	Class	Accuracy (%)
Ensemble SVM [26]	RR interval, HOS, wavelet, time domain, morphology	45	Inter-patient	5	94.5
Random Forest [25]	RR interval, HBF, time domain, morphology	6	Inter-patient	5	96.14
Naïve bayes [30]	HOS	4	Inter-patient	5	94
SVM [31]	RR-Interval, DCT Random projection	33	Inter-patient	5	93.1
Ensemble of BDT [32]	RR-interval, DCT random projection	33	Inter-patient	5	96.15
Ensemble SVM [33]	RR-Interval, Random projection	101	Inter-patient	5	93.8
Deep neural network [34]	RR interval, Wavelet, HOS, morphologcy	45	Inter-patient	4	89.25
This work (SVM)	RR interval	9	Inter-patient	5	92.57
This work (NN)	RR interval	9	Inter-patient	5	92.50
This work (RF)	RR interval	9	Intra-patient	5	96.22
This work (ANN)	RR interval	9	Intra-patient	5	**96.35**
This work (RF)	RR interval	9	Intra-patient(O)	5	**99.67**
This work (DT)	RR interval	9	Intra-patient(O)	5	**99.31**

**Table 18:** Experiment result on healthy person within 20 min.

	Average Processing Time (Second)	Found Beat					Number Beats	Average RSSI (dBm)
		N	S	V	F	Q		
RF	0.108739	1172	0	0	0	0	1172	−49.15
ANN	0.043825	1172	0	0	0	0	1172	−62.80
ANN-ROS	0.043033	1177	0	0	0	0	1177	−69.98
DT-ROS	0.000359437	1169	0	0	0	0	1169	−67.23
RF-SMOT	0.108851	1177	0	0	0	0	1177	−64.63
KNN-ROS	0.001943876	1171	0	0	0	0	1177	−52.47
RF-ROS	0.10563	1176	0	0	0	0	1176	−45.032

**Table 19:** Result the of experiment on six healthy people within 30 min.

Participant	Age	Gender	Found Beat					Average RSSI (dBm)
			N	S	V	F	Q	
1	33	M	1764	0	0	0	0	−63.7
2	34	M	1773	0	0	0	0	−59.1
3	36	M	1753	0	0	0	0	−59.3
4	35	M	1773	0	0	0	0	−46.9
5	28	F	1752	0	0	0	0	−72.8
6	33	F	1772	0	0	0	0	−60.5

## Data Availability

For training the classifiers, we use dataset from MIT-BIH Arrhythmia Database (https://physionet.org/content/mitdb/1.0.0/, accessed on 5 September 2021), the experiment data are available from the corresponding authors on reasonable request.
